# Unusually low infection rate of *Dirofilaria immitis* in its wildlife hosts by the northern border of the Mediterranean climate zone in Hungary

**DOI:** 10.3389/fvets.2025.1671338

**Published:** 2025-12-18

**Authors:** Eszter Nagy, Rebeka Ráhel Nagy, Ágnes Csivincsik, Tibor Halász, Sibusiso Moloi, Melinda Kovács, Gábor Nagy, Tamás Tari

**Affiliations:** 1 Institute of Wildlife Management and Wildlife Biology, Faculty of Forestry, University of Sopron, Sopron, Hungary; 2 Institute of Evolution, HUN-REN Centre for Ecological Research, Budapest, Hungary; 3 Centre for Eco-Epidemiology, National Laboratory for Health Security, Budapest, Hungary; 4 One Health Working Group, Kaposvár Campus, Hungarian University of Agriculture and Life Sciences, Kaposvár, Hungary; 5 Zselic Wildlife Estate, Somogy County Forest Management and Wood Industry Share Co., Kaposvár, Hungary

**Keywords:** red fox, golden jackal, European badger, *Dirofilaria immitis*, Hungary

## Abstract

Wildlife-originating zoonotic pathogens represent a special form of human-wildlife conflict. Disease spillover and spillback can cause health damage to both sides. Canine heartworm (*Dirofilaria immitis*) is considered a climate-sensitive parasite due to the special environmental demands of its mosquito vectors. Abundant wild mesocarnivores in Europe, the golden jackal (*Canis aureus*), the red fox (*Vulpes vulpes*), and Eurasian badger (*Meles meles*) are frequently accused of being a natural reservoir for the parasite. This study investigated the heartworm infection rate in the populations of jackals (*N* = 305), foxes (*N* = 361) and badgers (*N* = 29) by the northern border of the Mediterranean climate zone and, despite the suitable climatic conditions, found unusually low prevalence in these hosts (2.3% in jackals, 1.4% in foxes, 0% in badgers). Analysis of the spatial distribution of infection confirmed that temperature and local socioeconomic development influenced the disease occurrence significantly. Precipitation and land use did not show any impact on the epidemiology of canine heartworm in wild caniforms. These results suggested that wild carnivores are sentinels of *D. immitis* spilled over from the domestic cycle rather than vice versa.

## 1 Introduction

Zoonotic diseases at the domestic-sylvatic interface are special cases of human-wildlife conflicts. About 43% of zoonotic pathogens are estimated to originate from carnivore species (1). Due to their trophic characteristics, carnivores can carry more parasites than any other mammalian taxa; thus, they are frequently accused of playing the role of reservoirs or bridge hosts that carry zoonoses into the anthropogenic environment (2, 3). The disease transmission dynamics between different health domains need a multidisciplinary approach to analyse because mostly complex drivers contribute to the circulation of pathogens at the domestic-sylvatic interface. One Health concept, which exceeds the investigation of direct host-pathogen interaction, involves the cooperation of various fields of science for a better understanding of the background of endemics (4).

Canine heartworm (*Dirofilaria immitis*) is an indirectly developing zoonotic parasite. In its life cycle, wild and domestic canids, felids (1) and mustelids (5, 6), play the role of definitive hosts, providing a habitat for adult parasites. In humans, who can acquire infection by a mosquito bite, *D. immitis* causes heterogeneous symptoms due to the localisation of larval migration in the body and the patient’s immune response ability (1). On the other hand, mosquitoes serve as intermediate hosts, being responsible for transmission of the infection (7). Since mosquitoes’ breeding and survival depend mostly on weather conditions, *D. immitis* is considered a climate-sensitive parasite (8).

Global climate change is characterised by warming and increased occurrence of extreme weather events, such as heatwaves, heavy rainfalls, and stormwater floods. These extreme events are proven stressful conditions for living organisms, which result in the mitigation of immune response ability (9). Additionally, floods enhance the breeding success of floodwater mosquitoes, like *Aedes vexans* and *Aedes* (syn *Ochlerotatus*) *caspius* (10), which are confirmed to be important vectors of *D. immitis* in Europe (11, 12). Studies conducted recently in Hungary suggest that *Ae. vexans* shows an increased susceptibility to filaroid parasites (13, 14).

On the other hand, the European populations of the most abundant canid definitive hosts of *D. immitis* are also in expansion. In the case of the red fox (*Vulpes vulpes*), a successful vaccination campaign against rabies is presumed to be the background of the population growth during the last three decades (1, 15). The ongoing increase of the European golden jackal (*Canis aureus*) can be explained by several factors. Researchers found multiple causes that might have contributed to the rapid extension of the golden jackal across Europe. Change in climate and land-use, extreme population expansion of rodent species, and the lack of large carnivores might support the population growth and consequential migration westward (16).

However, the increasing trend of dog keeping might also enhance the risk of *D. immitis* infection. During the previous two decades, the number of pet dogs increased remarkably in Europe (17). The dense populations of domestic dogs provide available definitive hosts for the parasite to complete its life cycle. The presence of *D. immitis* is confirmed in the European domestic dog populations, with higher rates of infection among stray dogs (18). Large sample research in Hungary confirmed that prevalence of the parasite has been continuously rising since the first occurrence in 2009 (19). The potential risk-increasing effect of domestic dogs is worsened by the socioeconomic deprivation of a region. In regions of low-income human communities, the number of stray dogs and the lower level of disease prevention in owned dogs worsen the epidemiological situation (20, 21).

The urban environment might also play a role in the circulation of *D. immitis*. Besides the dense domestic dog populations, human settlements provide special conditions for both wild carnivores and mosquitoes. Communal wastes and abundant rodent populations mean more food, while green spaces and empty old buildings serve as shelters for wild mesocarnivores (22). The microclimate of the urban areas differs from their surroundings. The temperature is higher all year round, which correlates with lower humidity. Higher urban temperatures can contribute to the overwintering success of mosquitoes. Moreover, the warm environment can extend the breeding season of these insects. Though lower humidity decreases the survival rate of adult vectors, higher temperature supports longer and more frequent occurrence of mosquitoes in urban environments (23). Research in the southwestern part of Hungary close to our sampling area confirmed that *Ae. vexans*, a potential intermediate host for *D. immitis*, occurred in high abundance around human settlements, and their estimated infection rate by filaroid nematodes was higher than in habitats farther from human-populated areas (13).

The reservoir role of wild canids is under discussion. Studies in the early 2000s supported the hypothesis that wild carnivores can maintain the disease in the absence of spill-over from domestic dog populations (24). However, latest studies suggest that stray dogs and unprotected owned dogs can be the most important sources of parasite circulation within a hotspot (20, 21, 25). In a suitable environment, the sympatric presence of wild and domestic carnivores resulted in a shared parasite community, and both spillover and spillback mechanisms can enhance the disease transmission through the domestic-sylvatic interface, contributing to increased human health risk (2). The epidemiological importance of mustelids is questioned because only three cases of European badger are documented (5, 6).

The presence of mesocarnivores inside the settlements constitutes a conflict with the human community. Besides the risk of zoonoses, these animals are also charged with predation on domestic animals and littering by defecation and exploitation of street wastebins (15, 22). However, the anthropogenic environment and food source can cause health damage to wildlife, which enhances disease transmission between the connected health domains (26, 27).

This study aimed to determine the prevalence of *D. immitis* in the populations of red fox, golden jackals, and European badgers in the southwestern part of Hungary, and to identify the climatic, land cover, and socioeconomic factors, which might influence the occurrence of *D. immitis* in wild canid mesocarnivores in a region under a strong Mediterranean climatic impact. Our further aim was to collect evidence on whether wildlife hosts could be reservoirs or bridge hosts of *D. immitis* in the southwestern part of Hungary.

## 2 Materials and methods

### 2.1 Study site

In our study, conducted between October 2019 and February 2025, European badger (*n* = 29), red fox (*N* = 361), and golden jackal (*N* = 305) specimens were collected to assess the level of *D. immitis* infection in these hosts. The study area covered partly Somogy (46.4170 N, 17.583E) and Baranya (46.08333 N, 18.25E) Counties in the southwestern region of Hungary which are geographically closely attached to the Western Balkan region. The northern borders of the western Balkan region of Europe can be defined in various ways, for instance, the Trieste–Odessa line, the Sava, Krka, and Danube Rivers (28). Therefore, the geographic demarcation of the southern part of Hungary from the western Balkans could be difficult and based mostly on political issues (29). This part of the country is climatically and geographically closely attached to the western Balkan region of Europe (30). This geographic connection also means that there is an overlap between wild animal populations and their infectious diseases, e.g., alveolar echinococcosis (31, 32), fascioloidosis (33, 34) and bovine tuberculosis (35, 36), as has been confirmed by several studies.

A climate with strong Mediterranean influence characterises the sampling areas (37). On the south and the north, the Drava River and Lake Balaton border the study site, respectively. The catchment of Drava is part of the Mura Drava Danube Ecosystem Reserve, which is called “European Amazon” due to its humid and warm climate, and the dense vegetation of floodplain forests (38). Lake Balaton, covering a surface area of 593 km^2^, is the largest lake in Central Europe. However, the lake is an important tourist attraction in Hungary and the surrounding countries. Therefore, over half of the shoreline, approximately 128 km out of 240 km, is artificial (39). On the other hand, a special contiguous wetland habitat, the Nagyberek connects to the lake from the south (40). The South Transdanubia is a hilly area with diverse surface relief, high forest cover, and agricultural landscape. In the valleys, a high level of groundwater creates wet habitat patches (41, 42).

All investigated animals were legally hunted within the framework of the national hunting management programme. The animals were killed in accordance with the National Hunting Act (Act LV of 1996) and its implementing regulation (Ministry of Agriculture and Rural Development Decree 79/2004). The legislative background of our carnivore research is presented in detail at https://zenodo.org/records/15645626. After shooting, the carnivores were necropsied within 24 h. The cardiopulmonary systems were removed from the animals, and the heart and lung vessels were opened to detect the presence of *D. immitis* specimens. The collected worms were identified by their morphology (43).

### 2.2 Statistical analysis

Prevalence and mean intensity with the 95% confidence interval (CI 95%) were determined for the whole sample and separately for the fox and jackal populations. We then compared the two host species based on the aforementioned indices. The calculations were performed using the QPweb online application.^1^

**Figure 1:**
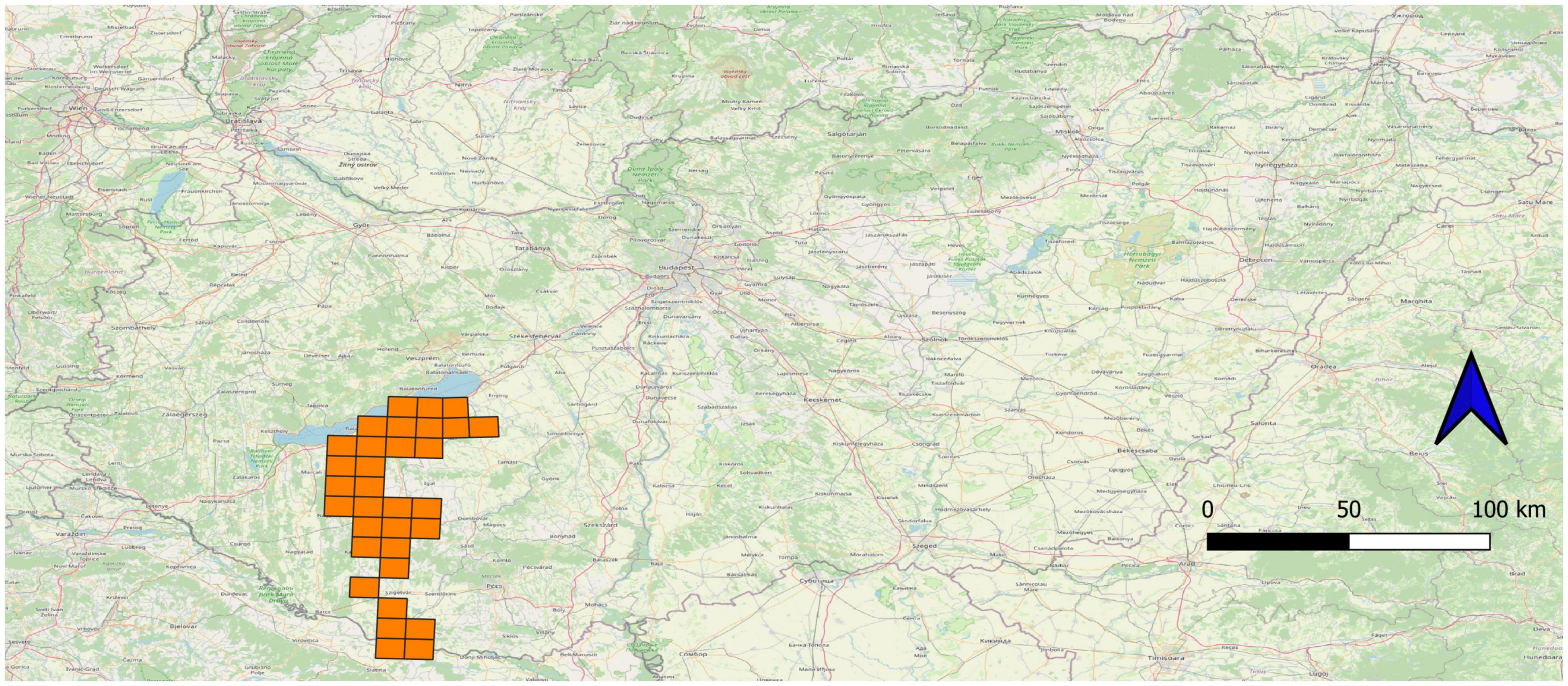
Position of the 10 × 10 km Universal Transverse Mercator (UTM) UTM grids of the studied area in Hungary, covering the coordinates of all samples included in the study (*n* = 695).

For further statistical analysis, we recorded the shooting coordinates of the animals. First, we mapped all shooting locations into the 10 × 10 km Universal Transverse Mercator (UTM) system. The prevalence of UTM grid cells was calculated based on the number of animals hunted in a particular UTM grid and the infected specimens (44) (Figure 1). The basic data of animal specimens were collected into files 1_Dirofilaria_immitis_animals.kml and 2_Dirofilaria_immitis_animals.csv in Zenodo repository at https://zenodo.org/records/17073351.

Using the calculated prevalences of the UTM grid cell to improve the crude rate of dirofilariosis, the Empirical Bayes smoothing technique was applied and recorded as EBSRATE. This smoothed rate was used as the dependent variable in a further analysis. For the visualisation of the EBSRATE, a thin plate spline interpolation was prepared. These steps were conducted using GeoDa software (version 1.22) and QGIS (version 3.16 Hannover).

In the present study, six potential environmental factors were selected to assess the risk of dirofilariosis in wild canids. They are separated into three categories, i.e., land cover, climate and socioeconomy. The choice of land cover categories is based on previous studies that have already confirmed that the different importance of each category could considerably affect the epidemiology of *D. immitis* (45, 46). We calculated the proportion of agricultural areas (AGRO), forests (FOREST), and the summarised area of wetland habitats and water bodies (WW) for the involved UTM grids. The Ecosystem Map of Hungary^2^ was used to classify the four land cover types.

The climatic characterisation of the UTM grid cells was carried out by the ClimatEU software (version 4.63). A seasonal limitation was applied to investigate the climatic factors. The larval development of *D. immitis* at daily mean temperatures below 14 degrees Celsius requires a longer period to emerge L3 stage larvae in intermediate mosquitoes (44). On the other hand, a finer, higher hourly temperature model revealed that the temperature fluctuation (19 ± 9 °C) can shorten the development period of the L3 larvae (47). Therefore, each grid was indicated by the mean temperature from May to September (Tm5-9) because the mean temperature usually exceeded the developmental threshold value. Regarding the total rainfall (PPTm5-9), we also characterised the UTM grid cells for this period. Both climatic variables were calculated over the 30-year (1991–2020) interval using the central coordinates of the UTM grid cells. Calculation of climatic factors based on file 5_Dirofilaria_immitis_climate_monthly-values_1991-2020.csv at https://zenodo.org/records/17073351.

The regional developmental level might also influence the risk of heartworm infection of local canids (20, 21); therefore, we used a socio-economic level variable (district development index, DDI) in our analysis. The data obtained from the Institute for Economic and Enterprise Research, Hungarian Chamber of Commerce and Industry and calculated from 21 different indicators.^3^ For calculation of DDI, we used socioeconomic factors collected in file 6_Dirofilaria_immitis_District_Development_Index.pdf at https://zenodo.org/records/17073351. The potential effect of the DDI was calculated by smoothing as described at EBSRATE calculation. After this process, we applied the calculated value of the central point of the UTM quadrant (DDI) in the model as an independent variable. For the visualisation of the DDI, a thin plate spline interpolation was prepared.

We used a generalised linear regression model with a Gamma distribution to assess the impact of the candidate explanatory variables. For model building, we used a dataset arranged in 3_Dirofilaria_immitis_factors.csv at https://zenodo.org/records/17073351. Before building the model, we check multicollinearity to avoid interfering correlation between the independent variables. If an explanatory variable had a greater variance inflation factor (VIF) than 2, we reject it from the model building. We applied such a low VIF to decrease the number of variables to an acceptable level for analysing 32 UTM quadrants. On one hand, the final model was accepted if the omnibus test showed a significant difference (*p* < 0.05) comparing the fitted model against the intercept-only model. On the other hand, the corrected Akaike’s information criterion (AICC) was used for model assessment. The lower the AICC, the better the fitness of the model.

## 3 Results

In total, 695 carnivore carcasses were dissected in the study, comprising 29 badgers, 361 foxes and 305 jackals. Infection was characterised by a 1.7% (CI95% = 1–3%) overall prevalence and a mean intensity of 2.83 (CI95% = 2–3.67) parasites per infected host for the whole sample. Five infected individuals were found among the foxes tested, seven among the jackals, and none in the badgers. Thus, the prevalence and mean intensity were 1.4% (CI95% = 0.5–3.2%), 2.4 (CI95% = 1.4–3.2) and 2.3% (CI95% = 1.1–4.47%), 3.14 (CI95% = 1.71–4.29) in foxes and jackals, respectively. None of these indices showed significant differences between the hosts (prevalence, *p* = 0.40; mean intensity, *p* = 0.38). Details can be seen at https://zenodo.org/records/17073351 (in file 4_Dirofilaria_immitis_QPweb_results.txt).

**Figure 2:**
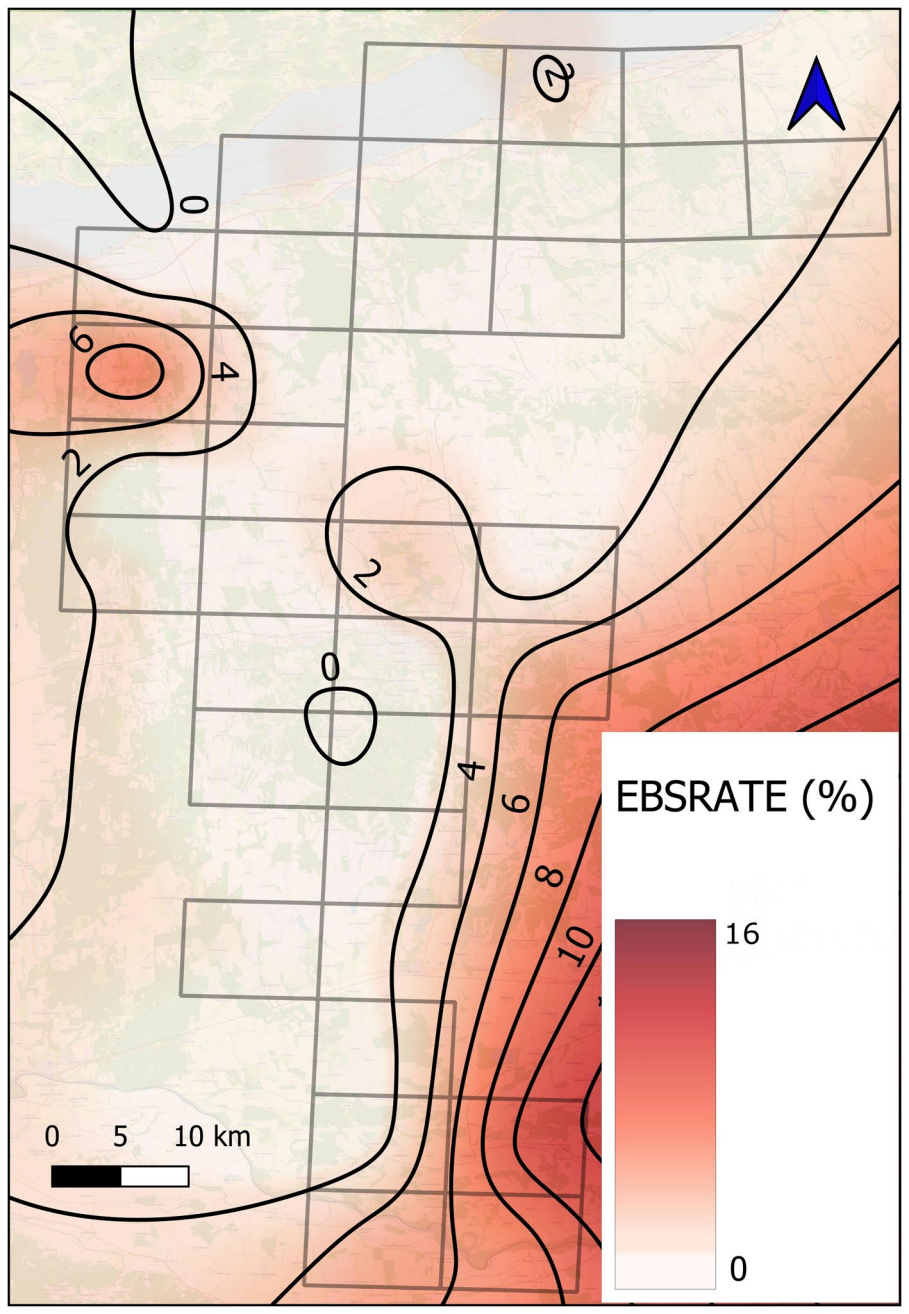
Visualisation of Empirical Bayes smoothed rate (EBSRATE) of canine dirofilariosis in fox and jackal populations in southwestern Hungary. The calculation was based on 666 fox and jackal specimens harvested between 2019 and 2025. The grid of straight lines represents the sampling UTM quadrats, while the sinuous lines indicate the levels of prediction. During visualisation, the smoothed prevalence was marked in 2% increments.

The Empirical Bayes smoothed rate (EBSRATE) of canine dirofilariosis prevalence showed heterogeneous dispersion across the study area. The mean of the predicted prevalence was 2.07% (CI95% = 1.23–2.92%). Higher *D. immitis* infection was concentrated in the southern part of the study area. Apart from some UTM squares blocked in the central and a few in the northwestern parts, the predicted prevalence showed a moderate and lower level in the rest of the study area (Figure 2).

After checking multicollinearity, four candidate variables were retained because variance inflation factor (VIF) value did not exceed the threshold value (VIF ≤ 2) (Table 1).

**Table 1:** Characteristics of the dependent and independent variables.

Variable	Unit	Range	Mean (SD)	Median	VIF
EBSRATE	%	0.33–10.16	2.06 (±2.03)	1.33	NA
AGRO	Proportion	0.09–0.74	0.47 (±0.16)	0.50	12.10
FOREST	Proportion	0.06–0.74	0,32 (±0.16)	0.34	2.31
WW	Proportion	0.01–0.72	0.10 (±0.17)	0.003	1.73
Tm5-9	°C	19.30–20.16	19.81 (±0.25)	19.88	1.25
PPTm5-9	mm	61.20–79.20	71.60 (±4.85)	72.30	1.58
DDI	Quintile	1.58–3.41	2.63 (±0.47)	2.7	1.8

For the final model, factors were selected based on their variance inflation factor (VIF). All explanatory variables were excluded from model building if their VIF value exceeded the specified threshold (VIF = 2). EBSRATE, Empirical Bayes smoothed rate of canine dirofilariosis; AGRO, proportion of agricultural areas; FOREST, proportion of forests; WW, proportion of summarised area of wetland habitats and water bodies; Tm5-9, average temperature from May to September in °C; PPTm5-9, mean precipitation from May to September in mm; DDI, district development index.

The regression model’s fitness was statistically significant compared to the null model (*χ*^2^ = 17.71, *p* = 0.01). The analysis revealed that in the best model (AIC = −188.26), only Tm5-9 and DDI had significant effects. Their regression coefficients suggested a positive and negative relationship between them and *D. immitis* infection level, respectively (Table 2). Nevertheless, the summarised extent of wetlands and surface water habitats (WW) had an OR of 3.08, though it did not prove significant (*p* = 0.32). The details of model building can be found at https://zenodo.org/records/17073351 (in file 7_Dirofilaria_immitis_SPSS_GLM_results.pdf).

**Table 2:** Results of the negative binomial regression analysis for the Empirical Bayes smoothed rate of canine dirofilariosis in foxes and jackals originated from the southwestern part of Hungary (2019–2025).

Predictor	*ꞵ*	*p*-value	OR
Intercept	−29.99	0.02	
WW	1.13	0.32	3.08
Tm5-9	1.32	0.04	3.75
PPTm5-9	0.03	0.45	1.03
DDI	−0.91	0.02	0.40

The level of significance was accepted in the cases when *p* < 0.05. EBSRATE: Empirical Bayes smoothed rate of canine dirofilariosis; WW: proportion of summarised area of wetland habitats and water bodies; Tm5-9: mean temperature from May to September in °C; PPTm5-9: mean precipitation from May to September in mm; DDI: district development index; *ꞵ* = regression coefficient; OR = odds ratio.

**Figure 3:**
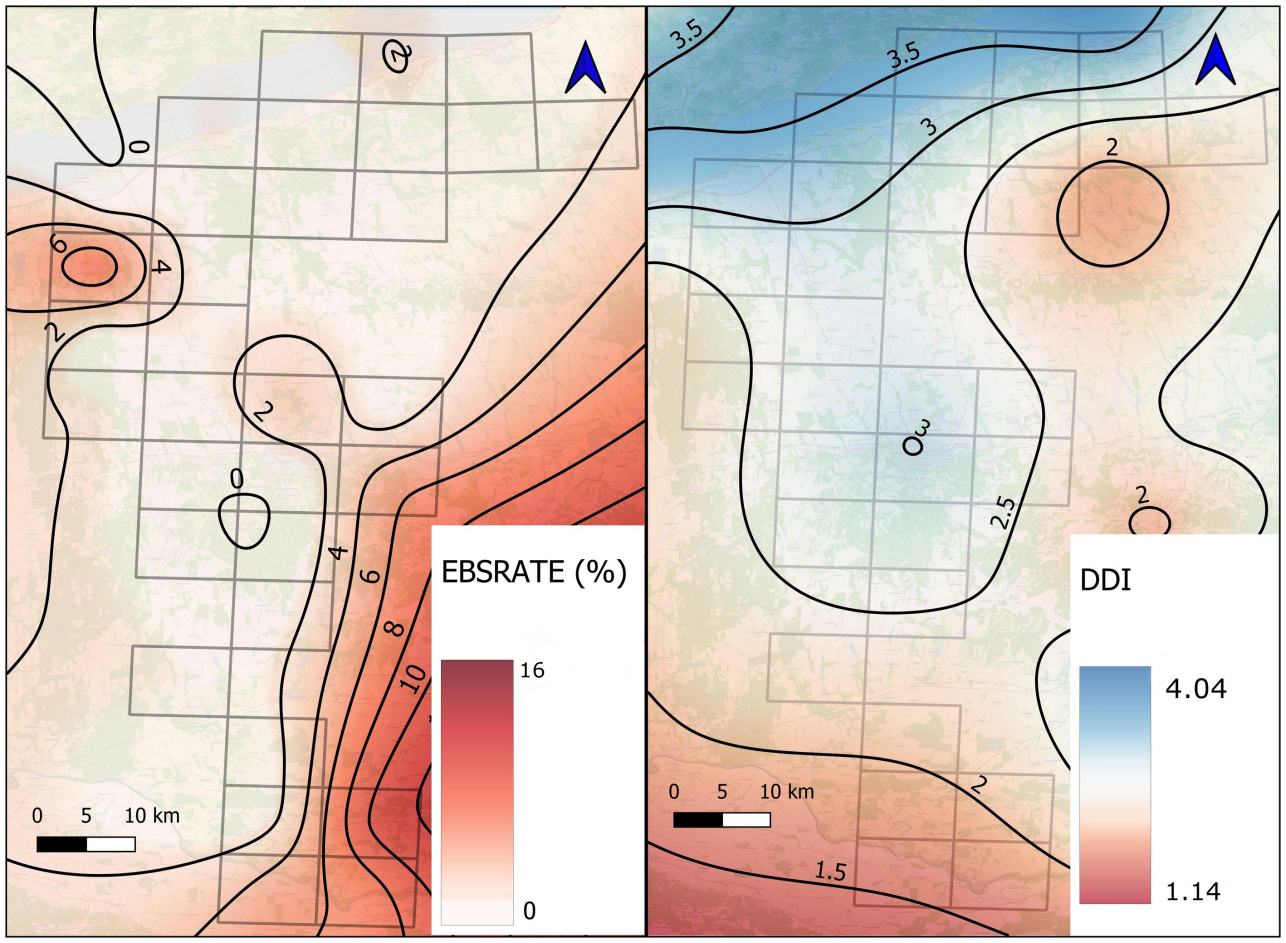
Demonstration of the relation between the Empirical Bayes smoothed rate (EBSRATE) of canine dirofilariosis and district development index (DDI) on the study area. The grid of straight lines represents the sampling UTM quadrats, while the sinuous lines indicate the smoothed prevalence of *D. immitis* infection in wild canids and the socioeconomic level. During the visualisation, the EBSRATE and the DDI were marked in 2 and 0.5% increments, respectively.

Comparing the spatial distribution of high-risk zones and low-income districts of the study area, UTM quadrants with the highest prevalence of infection were detected in the least developed districts of the study area. On the other hand, no case could be found in the most developed districts by the shoreline of Lake Balaton (Figure 3).

## 4 Discussion

Our study investigated heartworm infection of wildlife host populations of South Transdanubia, a region of Hungary with the strongest Mediterranean climatic impact. Despite the warm climate of the region and the humid microclimatic effect of extended forest stands and wet habitats, we found a low prevalence of *D. immitis* in both red fox and golden jackal, and none in European badger. The spatial analysis of sporadic cases of canine heartworm confirmed that temperature and regional socioeconomic deprivation positively influenced the occurrence of infection in wild canids. Nevertheless, we did not detect any effect of land cover features, and precipitation on the epidemiology of *D. immitis* in the study area.

Our findings on the prevalence of *D. immitis* in the two wild canid hosts contradicted the previously gained data on the parasite occurrence in wildlife in Hungary. Our study revealed a prevalence of 1.4 and 2.3% in foxes and jackals, respectively. Tolnai et al. (48) detected 3.7 and 7.4% during the investigation of 534 foxes and 27 jackals, respectively. Although the increasing health risk in dogs (25, 48) and in humans (49) has been confirmed in the country. Analysis of the spatial pattern of *Dirofilaria* spp. ascertained that the extension of canine heartworm endemic is more conspicuous in the eastern part of Hungary, while the spread of *D. repens* is witnessed in Transdanubia. The researchers identified increasing temperature as the most important risk factor, while precipitation proved less important (25).

Most researchers agree that global warming, thus extended breeding period of mosquitoes, contributes to the emergence of *D. immitis* in previously free northern areas (8). However, intermediate hosts of *D. immitis*, the mosquito species, need wet habitats for breeding and survival. Studies on the environmental demands of mosquitoes revealed that in the absence of suitable humidity, high temperatures can kill adult mosquitoes (23), and mild winters cause shorter diapause, thus increasing population loss in the spring (50). Considering that floodwater mosquitoes benefit from changing water levels of wetlands, it was surprising that our findings could not confirm the preliminary hypothesis that closeness of the two large water habitats (Drava River and Lake Balaton) of the study site increases the risk of dirofilariosis in wild canids. However, the map that visualised the Empirical Bayes smoothed rate (EBSRATE) of *D. immitis* infection in space (Figure 2) showed case accumulations both by the Drava River and Lake Balaton. The very low prevalence experienced in this study was not appropriate to confirm the risk-increasing effect of large surface water habitats.

Though the map illustration could show solely a suspicion on the epidemiological role of wet habitats within the study site, EBS rate of dirofilariosis in wild canids showed high values close to highly protected wetland habitats with contiguous canal systems, such as the area of the Old Drava Programme (51) and the Nagyberek by Lake Balaton (40). This finding highlights the potential role of wetlands in the epidemiology of *D. immitis*.

Moreover, we could not confirm risk-increasing effects of forest stands. However, authors agree that floodwater mosquitoes, especially *Ae. caspius*, are frequent insects of temporary waterholes in wooded areas (10). Therefore, we hypothesised that forests could also contribute to the maintenance and spread of canine heartworm. During model building, the forest coverage variable (FOREST) was excluded due to its high VIF. However, it should be noted that our study area is the most wooded region of Hungary, with 34.4% median forest coverage of the investigated UTM quadrants. The presence of water habitats and wetlands, areas where groundwater level can reach the soil surface at least once a year, is also characteristic for all investigated UTM quadrants with 0.3% median coverage. Therefore, we believe that the occurrence and breeding of floodwater mosquitoes might be expected at any point of the study sites.

In the case of the two climatic factors analysed, we found that temperature affected the smoothed rate of infection. It was curious that the local temperature during the mosquitoes’ main breeding period showed a significant impact on smoothed rate despite the narrow range between the coldest and warmest UTM quadrants. The odds ratio of this variable was 3.75 (*p* = 0.04), which represented a strong impact on infection. This finding called attention to an epidemiological risk-raising effect of the smallest temperature increase. On the other hand, the impact of precipitation proved negligible (OR = 1.03, *p* = 0.45); however, the range between the highest and lowest precipitation level is remarkable. Lower relevance of precipitation in the epidemiology of canine heartworm was also confirmed previously (25). Precipitation affects the survival and breeding success of mosquitoes (23). However, the macroclimatic features of the continental temperate zone provide enough precipitation for the occurrence of mosquitoes; thus, microfoci with higher humidity might not contribute excessively to increasing the risk of heartworm infection. Based on the findings on climatic factors of dirofilariosis, we concluded that a continuously high temperature, which assures a continuous presence of mosquitoes and stable conditions for larval development of heartworms, proved more relevant than precipitation, which can contribute to a denser mosquito population. Higher relevance of temperature above humidity is supported by the distribution pattern of *D. immitis* in Hungary. Eastern part of the country, the Great Plain, is characterised by extremely hot and dry summers. Despite apparently disadvantageous conditions for intermediate hosts, the prevalence of *D. immitis* in both mosquitoes (13, 52) and domestic dogs (19) is higher on the east than on the west (14). Data collected by investigation of mosquitoes in the westernmost area of the Balkans detected that only two of the sampled 1,015 mosquito pools proved positive for *D. immitis* resulting in 0.002% prevalence in intermediate hosts (13). In Serbia, Vojvodina bordering the Hungarian Great Plain, researchers detected 8.33% prevalence of *Dirofilaria* spp. in mosquitoes with 80 and 20% *D. immitis* and *D. repens* among them, respectively (52). This phenomenon is still to be explained; however, it agrees with our experiences.

The most curious finding of our research was the unusually low prevalence of *D. immitis* in both wild canid hosts in a region with strong Mediterranean climatic impact. This experience questioned the hypothesis that population expansion of the golden jackal enhances the distribution of canine heartworm (53). Moreover, the case accumulation in foci suggested that wild carnivores might be only sentinels of an infection spillover from a real reservoir. This hypothesis is partly supported by the findings of Naletilić et al. (54). They found 6.5 and 0% prevalence of *D. immitis* in the neighbouring Croatian wildlife by investigating 77 golden jackals and 326 red foxes, respectively. These values are the lowest in the Central European region. Therefore, the authors established that Croatian prevalence data cannot determine the role of wild carnivores in the epidemiology of *D. immitis* (54). The finding in European badger was not surprising since only two publications report cases in this host. Research in Romania detected one infected badger with an investigation of 115 individuals (5). Another study in Greece presented two separate clinical cases (6). Considering previous experiences together with our findings, the epidemiological relevance of mustelids is less probable, though it needs further research.

During the epidemiological analysis, we assumed that the domestic dog population of the region could serve as a reservoir of *D. immitis* infection. However, studies based on questionnaire survey (14) and blood sampling in veterinary facilities (19) proved that average prevalence in local dog population is 0.1–16.5%. Since our study was based on investigation of wild carnivores, we could not gain direct evidence on the role of dogs in the experienced phenomenon. However, the analysis of regional social deprivation confirmed a close correlation between the social vulnerability of the local human community and the infection risk of the sympatric wildlife (OR = 0.40, *p* = 0.02). The parallel delineation of the social variable and infection rate in wildlife visualises the interdependence (Figure 2). The heartworm-free status of the most developed districts by Lake Balaton suggested that the presence of wetlands alone could not increase the risk of infection in wildlife. This finding is supported by previous studies (20, 21), which confirmed that less developed districts remarkably contribute to the maintenance of *D. immitis* mostly by the presence of numerous unprotected domestic dogs.

The climatic impact of urban areas could not be confirmed by our data. Within our study area, only one big city, Kaposvár, exists. A mild EBSRATE increase could be detected north of the city (UTM quadrant YM14). The exact cause of this spot could not be revealed based on a few infected animals. Prevalence increase in the outskirts of large human settlements might have been due to a large population of less protected dogs (21) rather than the heat island of the city (23).

Our study aimed to reveal the infection rate of *D. immitis* in canid mesocarnivores of the southwestern part of Hungary with a climate under strong Mediterranean impact. We hypothesised that an optimal environment for potential mosquito hosts enhances the risk of *D. immitis* infection in natural hosts. However, we experienced unusually low prevalence in both red fox (5/361) and golden jackal (7/305) despite the large extent of suitable habitats in the study area. Investigating the risk factors of heartworm in wild carnivores, a strong impact of local temperature could be confirmed, while precipitation could not. Therefore, we concluded that the occurrence of mosquitoes, which depends on temperature, was more important in disease transmission than the density of mosquitoes, which is influenced by humidity. Moreover, a small increase in local temperature might increase the risk of infection remarkably.

The role of wetlands seemed important due to its odds ratio and the map illustration of the distribution of case accumulations; however, this apparent importance could not be confirmed statistically. In the background of this contradiction, we assume an anomaly, as most of the shoreline of Lake Balaton was free of *D. immitis* infected wild carnivores. These districts are the most developed ones within the study area; therefore, we concluded that the absence of numerous unprotected dog populations contributed to this advantageous epidemiological situation despite the extended mosquito habitats in the catchment of Lake Balaton. A completely different situation could be experienced by the Drava River. The case accumulation by the river was in one of the most deprived regions of Hungary, the Ormánság, where poverty affects a wide layer of the community (55). In this situation, the huge number of unprotected dogs might worsen the epidemiological status of wild carnivores.

Our study possesses limitations; therefore, it could not reveal the exact role of wild carnivores in the epidemiology of *D. immitis*, however, the reservoir role is less probable. We did not collect mosquito samples; thus neither the abundance nor the infection prevalence of intermediate hosts were determined. In the absence of these data, exact infection risk cannot be calculated. We did not survey domestic dog population of the area, and the knowledge, attitude and practice of the owners concerning heartworm. Therefore, we could not determine the true role of domestic dog population in the maintenance of dirofilariosis. However, the spatial analysis of the socioeconomic pattern of the study area suggested that wild carnivores, especially canids served as rather sentinels than reservoirs or bridge hosts of canine heartworm. Due to these limitations, our conclusion, especially in connection with socioeconomic aspects of diseases transmission, were based on deduction. Future research, which parallelly explores the infection rate in mosquito intermediate hosts, wild canids, and domestic dogs of the study area, could ascertain the disease dynamics at the domestic-sylvatic interface on the northern border of the Mediterranean climatic zone.

## Data Availability

The data that support the findings of this study are openly available in Zenodo at https://zenodo.org/records/17073351.

## Footnotes

1
https://www2.univet.hu/qpweb/qp10/index.php

2
http://alapterkep.termeszetem.hu/

3
https://gvi.hu/files/researches/608/GVI_JFM_2019_tables_en_190830.pdf
